# Cell-attached recordings of responses evoked by photorelease of GABA in the immature cortical neurons

**DOI:** 10.3389/fncel.2013.00083

**Published:** 2013-05-31

**Authors:** Marat Minlebaev, Guzel Valeeva, Vadim Tcheremiskine, Gaëlle Coustillier, Rustem Khazipov

**Affiliations:** ^1^Institut de Neurobiologie de la Méditerranée, Institut National de la Santé et de la Recherche Médicale U-901Marseille, France; ^2^Aix-Marseille UniversityMarseille, France; ^3^Laboratory of Neurobiology, Department of Physiology, Kazan Federal UniversityKazan, Russia; ^4^Laboratoire Lasers, Plasmas et Procédés Photoniques, UMR 7341 Centre National de la Recherche ScientifiqueMarseille, France

**Keywords:** GABA, patch-clamp, cell-attach, inhibitory postsynaptic potentials, uncaged GABA, RuBi-GABA

## Abstract

We present a novel non-invasive technique to measure the polarity of GABAergic responses based on cell-attached recordings of currents activated by laser-uncaging of GABA. For these recordings, a patch pipette was filled with a solution containing RuBi-GABA, and GABA was released from this complex by a laser beam conducted to the tip of the patch pipette via an optic fiber. In cell-attached recordings from neocortical and hippocampal neurons in postnatal days P2-5 rat brain slices *in vitro*, we found that laser-uncaging of GABA activates integral cell-attached currents mediated by tens of GABA(A) channels. The initial response was inwardly directed, indicating a depolarizing response to GABA. The direction of the initial response was dependent on the pipette potential and analysis of its slope-voltage relationships revealed a depolarizing driving force of +11 mV for the currents through GABA channels. Initial depolarizing responses to GABA uncaging were inverted to hyperpolarizing in the presence of the NKCC1 blocker bumetanide. Current-voltage relationships of the currents evoked by RuBi-GABA uncaging using voltage-ramps at the peak of responses not only revealed a bumetanide-sensitive depolarizing reversal potential of the GABA(A) receptor mediated responses, but also showed a strong voltage-dependent hysteresis. Upon desensitization of the uncaged-GABA response, current-voltage relationships of the currents through single GABA(A) channels revealed depolarizing responses with the driving force values similar to those obtained for the initial response. Thus, cell-attached recordings of the responses evoked by local intrapipette GABA uncaging are suitable to assess the polarity of the GABA(A)-Rs mediated signals in small cell compartments.

## Introduction

GABA is a main inhibitory neurotransmitter in the adult brain (Freund and Buzsaki, 1996; Farrant and Kaila, 2007). However, early in development, GABA, acting via chloride permeable GABA(A)-Rs, depolarizes immature neurons (Cherubini et al., 1991; Owens and Kriegstein, 2002; Ben Ari et al., 2007). The depolarizing GABA actions are due to an elevated intracellular chloride concentration as a result of weak functioning of the chloride extruder KCC2 and an enhanced activity of the chloride loader NKCC1 in immature neurons (Kakazu et al., 1999; Rivera et al., 1999; Payne et al., 2003; Yamada et al., 2004; Dzhala et al., 2005; Brumback and Staley, 2008; Blaesse et al., 2009). Depolarizing GABA actions were also observed in a number of other physiological and pathological conditions. These include depolarizing GABA actions in the axon initial segment (Szabadics et al., 2006; Khirug et al., 2008; Woodruff et al., 2011) and dendrites (Gulledge and Stuart, 2003) of adult pyramidal neurons and interneurons (Martina et al., 2001; Chavas and Marty, 2003), transient depolarizing GABA actions as a result of activity-dependent changes in ionic driving forces (Raimondo et al., 2012), as well as following trauma, in epilepsy, brain ischemia and pain (van den Pol et al., 1996; Cohen et al., 2002; Nabekura et al., 2002; Khalilov et al., 2003; Toyoda et al., 2003; Pond et al., 2006; De Koninck, 2007; Shulga et al., 2008; Price et al., 2009; Boulenguez et al., 2010; Dzhala et al., 2010, 2012). Depolarizing GABA actions are also subject to hormonal modulation, e.g., by oxytocin, which suppresses depolarizing GABA responses at birth (Tyzio et al., 2006; Mazzuca et al., 2011).

Different experimental approaches exist to detect depolarizing actions of GABA. The main requirements for these are: (1) an unperturbed intracellular chloride concentration and the reversal potential of the currents through GABA receptors (*E*_GABA_) and (2) an unperturbed resting membrane potential (*E*_m_). To achieve these conditions, several techniques have been developed including gramicidin perforated patch (Abe et al., 1994; Reichling et al., 1994), extracellular recordings of the local field potential (Glickfeld et al., 2009; Bazelot et al., 2010), multiple/single unit responses to GABA (Khazipov et al., 1997; Valeeva et al., 2010), cell-attached recordings of single GABA channels (Curmi et al., 1993; Serafini et al., 1995; Tyzio et al., 2006), cell-attached recordings of potassium or NMDA channels as voltage sensors (Zhang and Jackson, 1995; Leinekugel et al., 1997; Verheugen et al., 1999), cell-attached current-clamp recordings (Perkins, 2006), imaging of intracellular calcium transients evoked by GABA (Connor et al., 1987; Marty and Llano, 2005), intracellular chloride sensors (Kuner and Augustine, 2000; Marandi et al., 2002; Glykys et al., 2009), and voltage sensitive dye imaging (Wei et al., 2013).

Cell-attached recordings of GABA channels enable one to assess the polarity (depolarizing or hyperpolarizing) of GABAergic responses in a small patch of membrane from a single neuron (Curmi et al., 1993; Serafini et al., 1995; Tyzio et al., 2006). In this approach, GABA is added to the pipette solution at micromolar concentration to maintain the activity of 1–2 channels in the recorded patch of membrane, and the driving force acting on currents via GABA(A) channels (*DF*_GABA_ = *E*_GABA_ − *E*_m_) is estimated from the reversal potential of the currents through GABA channels. Although efficient, this technique has certain limitations, including eventual contamination of non-GABA channels in the recorded patch of membrane that are hard to control because with this technique, recordings from the same patch of membrane without GABA are unavailable [unless GABA is infused into the tip of the pipette (Curmi et al., 1993)]. In addition, currents via GABA channels show poor signal/noise ratio near the reversal potential (which is often <10 mV from the resting membrane potential) and thus the polarity of the GABAergic signals and *DF*_GABA_ values are deduced from approximations of the current-voltage relationships basing on values distant from the reversal potential. Here, we have attempted to overcome these problems by using photo uncaging of GABA during cell-attached recordings. Basing on previous studies using local GABA uncaging from RuBi-GABA in whole-cell recordings (Rial Verde et al., 2008), we envisaged that this would enable: (1) the generation of a fast pulse of GABA in the pipette solution to evoke an integral current in the recorded patch of membrane, detectable at the resting membrane potential despite small *DF*_GABA_ values; (2) the measurement of *DF*_GABA_ directly from the current-voltage relationships of the integral currents activated by uncaged GABA after the subtraction of the patch conductance prior to GABA uncaging. Because GABA needs to be released within the pipette, we have developed a device to provide laser pulse from inside the patch-pipette using a thin optic fiber introduced into the neck of the patch pipette. We show that intrapipette GABA photo-uncaging from RuBi-GABA rapidly activates tens of GABA(A) channels during cell-attached recordings that generate, at resting membrane potential, detectable integral GABA(A)R mediated currents. In neonatal rat cortical neurons, these initial responses are inwardly directed, indicating depolarizing GABA action, and their polarity is inversed to hyperpolarizing after the addition of the NKCC1 blocker bumetanide. The direction of these initial currents thus enables on the conclusion of whether GABA exerts a depolarizing or hyperpolarizing action on the recorded cell and, in this regard, the technique can be of interest to assess the depolarizing/hyperpolarizing actions of GABA in a variety of conditions when the polarity of GABA responses is a subject of investigation.

## Materials and methods

### Ethical approval

All animal-use protocols conformed to the guidelines of the French National Institute of Health and Medical Research (INSERM) and of the Kazan Federal University on the use of laboratory animals.

### Brain slice preparation

Acute coronal brain slices were prepared from P2-5 Wistar rats. The animals were cryoanesthetized, and the brain was rapidly removed to oxygenated (95% O_2_–5% CO_2_) ice-cold (2–5°C) artificial cerebrospinal fluid (ACSF) of the following composition (in mM): NaCl 126, KCl 3.5, CaCl_2_ 2, MgCl_2_ 1.3, NaHCO_3_ 25, NaH_2_PO_4_ 1.2, and glucose 11 (pH 7.4). Five hundred μm thick coronal slices were cut using a Vibratome (VT 1000E; Leica, Nussloch, Germany). Slices were kept in oxygenated ACSF at room temperature (20–22°C) for at least 1 h before use. For recordings slices were placed into a conventional submerged chamber and superfused on both sides with oxygenated ACSF at 30–32°C at a flow rate of 2–4 ml/min.

### Electrophysiological recordings

Patch-clamp recordings were performed using Axopatch 200B (Axon Instruments, Union City, CA, USA). Patch electrodes were made from borosilicate glass capillaries (GC150F-15, Harvard Apparatus, Edenbridge, UK). Patch electrodes had a resistance of 6–8 MOhms when filled with the pipette solution for cell-attached recordings contained (in mM): NaCl 120, TEA–Cl 20, KCl 5, 4-aminopyridine 5, CaCl_2_ 0.1, MgCl_2_ 10, glucose 10, Hepes–NaOH 10 buffered to pH 7.2–7.3. This solution differs from ACSF composition and introduces two sources of error in the estimation of the *DF*_GABA_: (1) a liquid junction potential of −2 mV and (2) a Goldman-Hodgkin-Katz (GHK)—error, which is the difference in the GHK voltage equation values obtained with anion concentrations of the pipette solution and of the extracellular fluid of −4 mV. Together, this gives an estimated *DF*_GABA_ value that is 2 mV more negative than the real *DF*_GABA_ value. *DF*_GABA_ values were not corrected for this error. GABA(B) receptor antagonists were not added to the pipette solution because postsynaptic GABA(B) receptors are little expressed in the neonatal cortical neurons (Gaiarsa et al., 1995); however, in older animals addition of the GABA(B) receptor antagonists to the pipette solution could be suggested. RuBi-GABA was added to the solution on the day of experiment at a concentration of 20 μM from a frozen 20 mM stock solution. Following the formation of a gigaseal, the gain was set to 50 mV/pA and the headstage was switched to the capacitor feedback mode. Recordings were performed using 2.7 s long sweeps (with a minimal inter-sweep interval) with the following voltage waveform at the pipette (*Vp*): (1) 0 mV for 1 s; (2) step to +55 mV for 100 ms; (3) 500 ms ramp from +55 mV to −55 mV; (4) holding at −55 mV for 100 ms; (5) 500 ms ramp from −55 mV to +55 mV; (6) 500 ms step to 0 mV. In the experiments on the current-voltage relationships of the initial part of the response, *Vp* was set at 0, +50, or −50 mV. Following stabilization of the resistance (typically within 5–10 sweeps), analogous output of a 1 s long voltage command was sent to trigger a 20–60 mW laser pulse. Five to ten sweeps were recorded following the pulse. In some cells, single GABA channels were then recorded at different holding potentials for further analysis of current-voltage relationships. The signals were digitized at 10 kHz using an analogue-to-digital converter Digidata 1440 (Axon Instruments, Union City, CA, USA).

### RuBi-GABA uncaging

Since RuBi-GABA is light-sensitive, special caution was taken to prevent its untimely uncaging with ambient light (Rial Verde et al., 2008). All experiments were performed at minimal ambient light in the experimental room. The computer screen was set at red mode. The filling of the patch pipette with internal RuBi-GABA containing solution, insertion of the pipette into the holder, positioning of the pipette, and establishment of a gigaseal were performed under a red filtered light.

Continuously radiating (cw) lasers with analog modulation of output power, models Spectra-Physics 161c-030 (30 mW, 488 nm) and DragonLazers (100 mW, 473 nm) were used as a light source for GABA uncaging. The laser beam was introduced into a multimode 50 μm optic fiber in core via a collimating lens, meeting the condition for the numerical aperture of the fiber (0.22 NA). The open end of the fiber was cut under the binocular loupe by a diamond, pulling the free end of the fiber with forceps. The nominal power of the output laser beam used in the experiment was ~20 mW. It was typically attenuated at the optic fiber output to 60–80%. The nominal light pulse duration used for uncaging was 1 s. A hole (about 0.3 mm in diameter) was drilled using a conic bid in the patch pipette holder to introduce the optic fiber into the patch pipette holder. The hole was then filled with dental cement to secure the fiber and to prevent air leakage. We kept the optic fiber 2–5 mm longer than the signal wire so that when the patch pipette was introduced into the holder, the fiber appeared in the neck of the patch pipette.

### Analysis

pCLAMP 10.1 (Axon Instruments, USA), Origin 7.0 (Microcal Software, Northampton, MA, USA) and custom-written functions in Matlab (MathWorks, MA, USA) were used for data acquisition and analysis. For each response, we calculated a slope of the initial response within a time window of 25 ms after the laser pulse onset. Current-voltage relationships of the integral response were analyzed after subtraction of the control ramp response in two directions as described above. After the decay of the response to a few (1–3) levels of GABA channels, in some cells, current-voltage relationships of the currents through single GABA channels were analyzed as described previously (Tyzio et al., 2006, 2008).

Group measures are expressed as means ± SE, and error bars also indicate SE. Data were assessed for normality using the Shapiro–Wilk test. The statistical significance of differences for normally distributed data was determined with the Student's *t*-test (paired and unpaired). The Wilcoxon Signed Rank test was used to compare non-normally distributed data. Unless indicated, the level of significance was set at *P* < 0.05.

### Drugs

RuBi-GABA was purchased from Tocris (Tocris Cookson Inc., USA). A 20 mM RuBi-GABA stock solution was prepared in the dark, divided in aliquots, and stored frozen in tightly sealed vials in a light-impermeable box. Other reagents were from Sigma (Sigma-Aldrich Inc., USA) and Tocris (Tocris Cookson Inc., USA).

## Results

**Figure 1:**
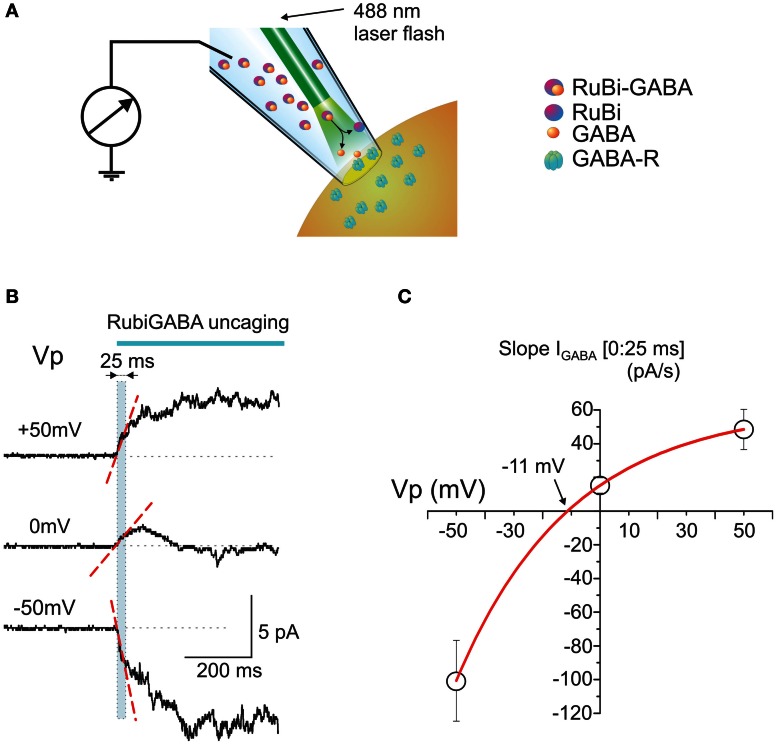
**Intrapipette GABA photorelease from RuBi-GABA evokes currents in cell-attached recordings. (A)** Scheme of the experimental setup. The patch-pipette is filled with a solution containing RuBi-GABA (20 μ M). An optic fiber is positioned close to the tip of the pipette. Once a gigaseal is formed between the pipette and neuronal membrane, cell-attached recordings enable the recording of currents through the ion channels in the patch of cell membrane attached to the tip of the pipette. After recordings of the baseline activity, a laser pulse is delivered via an optic fiber to uncage GABA from RuBi-GABA and to activate GABA(A) receptor-channels (GABA-Rs). **(B)** Example traces of the responses evoked by GABA uncaging in cell-attached recordings from P2-5 cortical neurons at different pipette potentials. Note that the initial response slope (indicated by dashed red line in a [0:25 ms] time window after the laser pulse onset outlined by cyan box) is positively directed (in cell-attached recordings the inward currents are upward) at the resting membrane potential (*Vp* = 0 mV), that it increases with patch hyperpolarization and changes its direction from depolarizing to hyperpolarizing during patch depolarization. **(C)** Plot of the dependence of the initial response slopes on the pipette potential. Data points show mean values ± S.E. Slope-voltage relationships are approximated with an exponential function which shows reversal near −11 mV that corresponds to *DF*_GABA_ of +11 mV.

**Figure 2:**
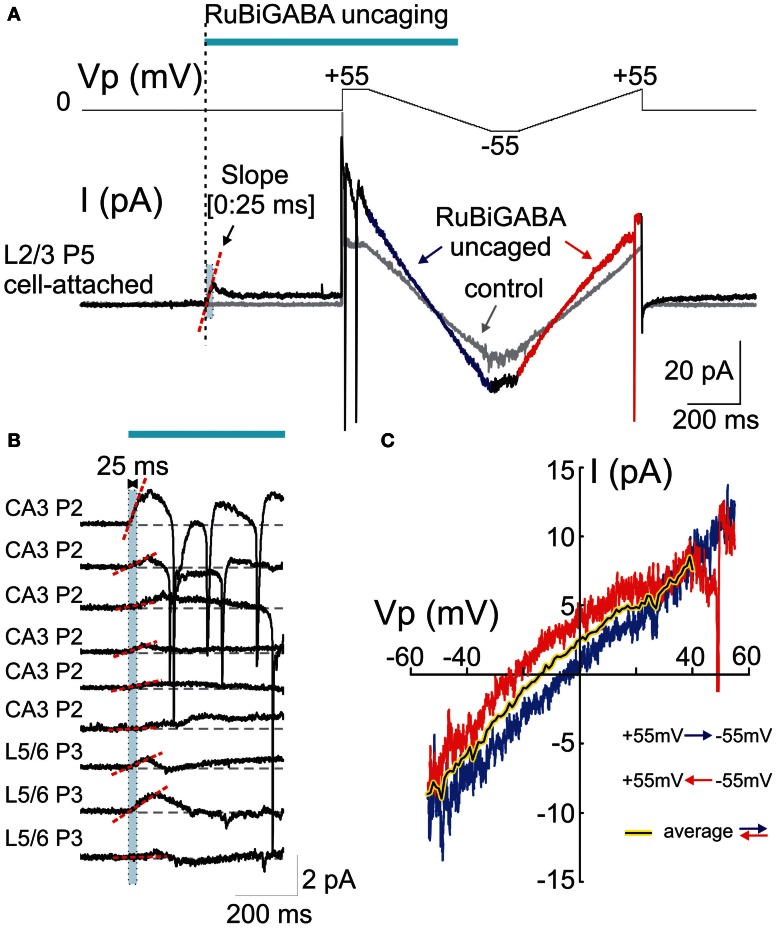
**Cell-attached responses evoked by photoreleased GABA and their voltage-dependence. (A)** Example trace of cell-attached recordings of currents evoked by GABA photorelease from a P5 rat L2/3 neuron. The voltage protocol is shown in the top trace and the cell-attached current is on the bottom trace. The pipette potential (*Vp*) is first held at 0 mV during GABA uncaging by a laser pulse. GABA uncaging evokes an initial inward current. Five hundred ms after the pulse onset, two-direction voltage ramps (between +55 and -55 mV; ramp directions are color coded) reveal an increase in the membrane conductance compared to the control trace before GABA uncaging (gray traces). **(B)** Examples of the initial cell-attached responses to RuBi-GABA uncaging at resting membrane potential (*Vp* = 0 mV) in hippocampal (CA3) and cortical (L5/6) neurons of P2-3 rats. Initial (0:25 ms) slopes are indicated by red dashed lines. Note the inward direction of the initial response slopes in all recorded cells. **(C)** Current-voltage relationships of the currents activated by uncaged GABA, obtained after subtraction of control ramps from the responses after RuBi-GABA uncaging during hyperpolarizing-to-depolarizing ramps (blue) and depolarizing-to-hyperpolarizing ramps (red). Note a difference in the reversal potentials of these curves that indicates a hysteresis of the GABA conductance reversal potential. The black-on-yellow curve is a smoothed average of the current-voltage relationships obtained during bidirectional ramps.

Cell-attached responses to photo-uncaged GABA from Rubi-GABA were studied in cortical and hippocampal neurons of neonatal P2-5 rats. Patch pipettes were filled with a solution containing Rubi-GABA (20 μ M) and the laser pulse for GABA uncaging was provided by an optic fiber introduced into the patch pipette (Figure 1A). Typical responses to GABA uncaging at different potentials of the recording pipette (*Vp*) are shown on Figure 1B. At the holding potential of 0 mV (which corresponds to the resting membrane potential) the response was characterized by an initial inwardly directed current (note that in cell-attached recordings the inward currents are upward; see also Figure 2B). Because the whole-cell GABAergic currents activated by RuBi-GABA photo-uncaging attain peak values within the first tens of milliseconds [28 ms for 5 ms pulses (Rial Verde et al., 2008)], we analyzed the initial response slope during the first 25 ms after the laser pulse onset (the region outlined by the dashed cyan box in Figure 1B). The initial response slope is determined by the direction and magnitude of current through the GABA(A) receptors, and thus reflects the number of open channels and the driving force acting on ions flowing through GABA(A) channels (*DF*_GABA_) at the response onset. In its nature, this parameter is similar to the slope of extracellularly recorded GABAergic postsynaptic currents (Glickfeld et al., 2009; Bazelot et al., 2010) with a difference that it is generated by several open GABA(A) channels in the recorded patch of membrane. Because *DF*_GABA_ is highly use-dependent, the initial response slope provides a valuable measure of the polarity of the response to GABA in an unperturbed neuron.

**Figure 3:**
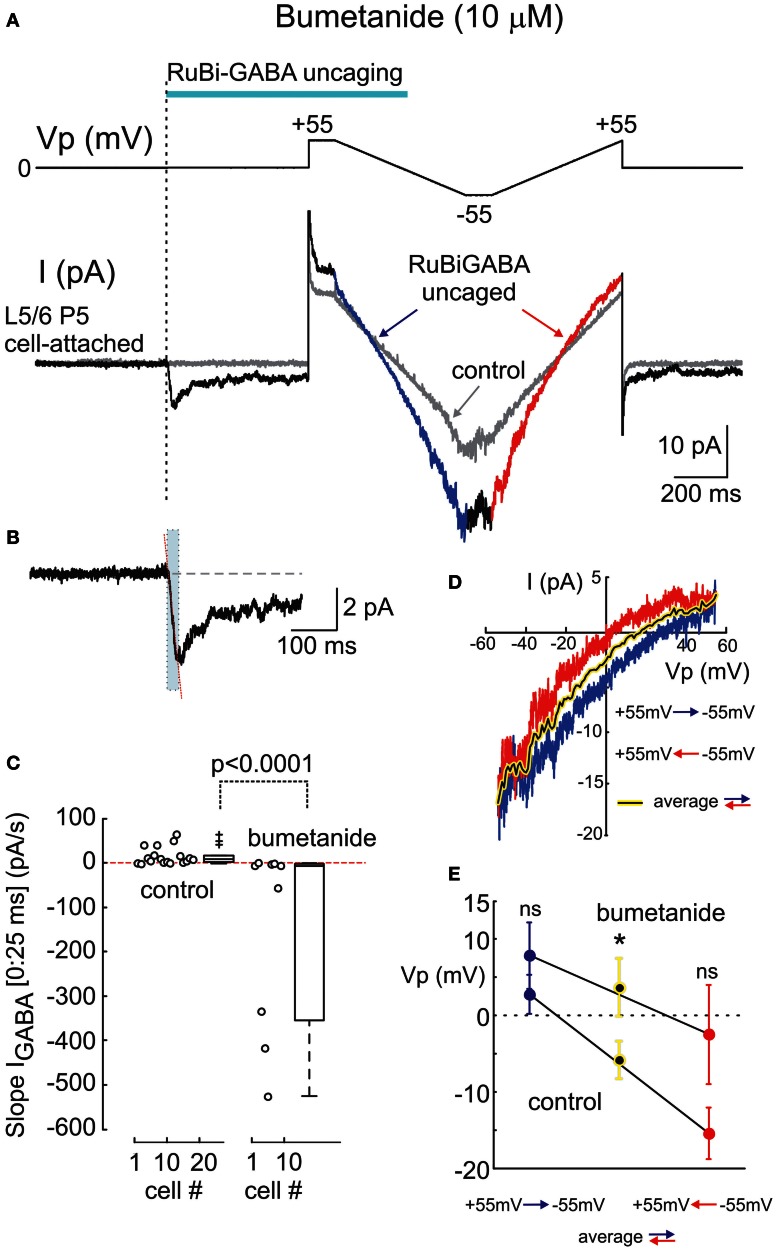
**Effects of the Na^+^-K^+^-2Cl^−^ co-transporter bumetanide on cell-attached responses evoked by uncaged GABA. (A)** Example responses to GABA uncaging in cell-attached recordings at resting membrane potential from a P5 rat L5/6 neuron in the presence of bumetanide (10 μM). Layout is the same as on Figure 2A. The initial response to uncaged GABA on an expanded time scale is shown in panel **(B)** where the dashed line indicates an outwardly directed (0:25 ms) initial slope. **(C)** Summary plot of the initial (0:25 ms) slope values at resting membrane potential in control conditions and in the presence of bumetanide (10 μ M). Each circle corresponds to an individual cell. Boxes indicate 25–75% confidence intervals. Note that the initial slopes change direction from inward to outward after the addition of bumetanide. **(D)** Current-voltage relationships of the uncaged GABA-activated cell-attached currents during bidirectional ramp voltage commands (layout is same as in Figure 1C). **(E)** Summary plot of the reversal potentials of the uncaged GABA activated currents using the ramp protocol. Blue and red circles (mean ± S.E.) correspond to the hyperpolarizing-to-depolarizing and depolarizing-to-hyperpolarizing ramp directions, respectively. Black on yellow circles correspond to the bidirectional ramp averages. **(C–E)** Pooled data from 16 neurons in control conditions and 9 neurons in the presence of bumetanide (P2-5 rats; ^*^p < 0.05; ns, nonsignificant).

At the resting membrane potential (*Vp* = 0 mV), the initial slopes ranged from -1.9 to 64.1 pA/s (mean ± *SE*: 15.2 ± 4.6 pA/s; *n* = 18 cells; Figures 1B,C, 2B, 3C). Initial slope values revealed depolarizing responses both in P2-5 neocortical neurons (15.7 ± 5.6 pA/s; *n* = 11 cells) and CA3 hippocampal neurons (14.5 ± 8.5 pA/s; *n* = 7 cells). These values were not significantly different (*P* > 0.05) and therefore the data obtained from the neocortical and hippocampal neurons were pooled together during analysis. The positive direction of the currents evoked by uncaged GABA indicates a depolarizing action of GABA in these neurons, which is consistent with results obtained using other approaches (see Introduction).

**Figure 4:**
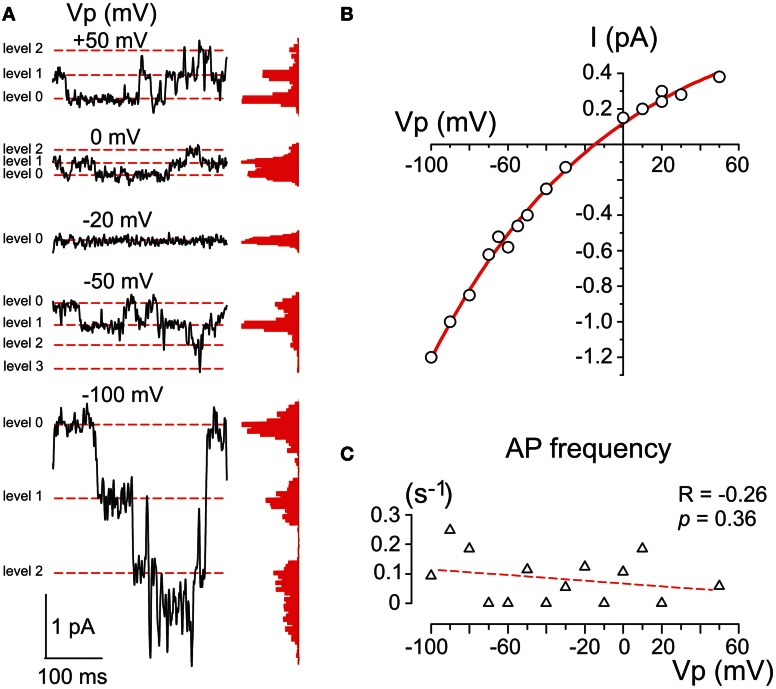
**Single GABA channels activated by photorelease of GABA. (A)** Example traces of cell-attached currents through GABA channels activated on the decay of the response evoked by RuBi-GABA uncaging at different holding potentials of the recording pipette. Corresponding all current point histograms are shown on the right from each trace. Levels are indicated by red dashed lines. Data from a P2 CA3 pyramidal cell. **(B)** Current-voltage relationships of the amplitudes of currents through single GABA channels activated by uncaged GABA in the cell shown in panel A. An exponential fit of the current-voltage relationship shows a reversal at -15 mV that corresponds to a *DF*_GABA_ value of +15 mV. **(C)** Dependence of the action potential frequency on *Vp*. Note that the cell does not fire more with an increase in *Vp*.

The slopes of the initial response to GABA uncaging were compared at different holding potentials of the recording electrode: hyperpolarized (+50 mV) and depolarized (−50 mV; Figures 1B,C). The initial depolarizing response (0:25 ms) slope increased during 50 mV membrane hyperpolarization to 48.5 ± 12.1 pA/s (*n* = 4), but inverted its polarity during membrane depolarization attaining values of −100.6 ± 24.0 pA/s at *Vp* = −50 mV (*n* = 3) (Figure 1C). An exponential fit of the initial current slope—voltage relationships revealed a reversal potential near −11.3 mV (Figure 1C), corresponding to depolarizing *DF*_GABA_ values of +11.3 mV, which is close to the value obtained during analysis of cell-attached recordings of single GABA channels at the tail of GABA-uncaging evoked response (see below, Figure 4) and with GABA continuously present in the pipette (Tyzio et al., 2006, 2008).

Depolarizing GABA responses in the immature cortical neurons are primarily maintained by NKCC1 chloride co-transporter (Payne et al., 2003; Yamada et al., 2004; Dzhala et al., 2005; Tyzio et al., 2006). Therefore, we next tested whether the depolarizing responses evoked by uncaged GABA depend on NKCC1 activity. In the presence of the bath applied selective NKCC1 blocker bumetanide (10 μM), the initial response to uncaged GABA changed its polarity to outward (Figures 3A,B) and the initial (0:25 ms) slope obtained an average of −144.4 ± 72.2 pA/s (range from −526.5 to −0.03 pA/s; *n* = 9 cells, pooled data from 6 neocortical and 3 hippocampal neurons) (Figure 3C). The outward direction of the initial response indicates that when the NKCC1 co-transporter is blocked, responses to GABA become hyperpolarizing, which is consistent with previous observations using cell-attached recordings of GABA activated channels (Tyzio et al., 2006, 2008). Thus, it appears that the direction of the photoreleased GABA-evoked initial response provides a reliable assessment of the polarity of GABA responses, as it shows a depolarizing direction in P2-5 cortical neurons and it is inverted to hyperpolarizing after the addition of bumetanide.

In the same experiments, we attempted to assess *DF*_GABA_ values from the voltage-dependence of the currents activated by photoreleased GABA using two-direction voltage ramps (from +55 mV to −55 mV) delivered 500 ms after the laser pulse onset (Figure 2A). Current-voltage relationships of the uncaged GABA activated conductance were analyzed after the subtraction of the control ramp-responses performed prior to photostimulation from the ramps obtained during the uncaged GABA-evoked responses. These revealed a conductance activated by uncaged GABA of 194 ± 52 pS (range from 35 to 750 pS) in the range of *Vp*-values from 0 to −50 mV (*n* = 16 cells; average of responses in both directions). Basing on a single GABA channel conductance of 11.3 pS (see below), we estimated that from 3 to 66 GABA channels (mean, 17 ± 5) contribute to the integral response during the ramp commands. As shown in Figures 2A,C, during ramps performed in a direction from hyperpolarized to depolarized membrane potentials (from +55 to −55 mV), uncaged GABA-activated currents reversed at a *Vp* of −2.7 ± 2.5 mV (which corresponds to *DF*_GABA_ of +2.7 mV), yet when the ramps were performed in a direction from depolarized to hyperpolarized membrane potentials, they reversed at *Vp* = −15.4 ± 3.4 mV (*DF*_GABA_ = +15.4 mV; *n* = 16 cells; *p* < 0.01; see also Figure 3E). Thus, the responses to uncaged GABA show a voltage-dependent hysteresis of their reversal potential, characteristic of the chloride conductance (Huguenard and Alger, 1986; Thompson and Gähwiler, 1989; Raimondo et al., 2012). In order to minimize the impact of hysteresis, the ramps going in both directions were averaged, and this revealed a reversal potential of the currents activated by uncaged GABA at *Vp* = −5.8 ± 2.5 mV (*DF*_GABA_ = +5.8 mV; *n* = 16; Figure 3C). A similar analysis of the current-voltage relationships of the currents activated by uncaged GABA using two-direction voltage ramps in the presence of bumetanide (10 μ M) revealed a tendency to shift the reversal potential during both hyperpolarization—to—depolarization (7.9 ± 4.4 mV) and during depolarization—to—hyperpolarization (−2.5 ± 6.5 mV) ramp directions, but was not significant (*n* = 9 cells; *p* > 0.05) (Figure 3D). Reversal potential of the averaged current-voltage relationships in both directions also shifted to more negative values of 3.7 ± 3.8 mV (compared to the control values of −5.8 ± 2.5 mV; *p* < 0.05) (Figures 3D,E). The changes in the initial response polarity and the negative shifts in the reversal potential of the uncaged GABA activated currents observed in the presence of bumetanide are consistent with the hypothesis that the depolarizing action of GABA in immature neurons is supported by NKCC1 activity. However, the *DF*_GABA_ values obtained during voltage ramp protocols, although depolarizing and bumetanide-sensitive, were more negative than those obtained using an analysis of single GABA channels at P2-5 [around +14 mV (Tyzio et al., 2006, 2008), see also below] and were affected by the voltage-dependent hysteresis of the reversal potentials. Moreover, many cells fired action potentials during voltage ramp protocols when the *Vp*-values were positive, independently of the ramp direction after (but not before) GABA uncaging (Figure 2A), and some cells fired action potentials after the initial response even at *Vp* = 0 mV (Figure 2B). This suggests an electrical access to the recorded cell via a large number of GABA channels activated by uncaged GABA [see also (Alcami et al., 2012)]. Small shifts in chloride gradient across the patch, gain of the electrical access to the cell, as well as activation of the voltage-gated conductances may be involved in the variability of current waveforms seen during the first 500 ms after RuBi-GABA uncaging (Figure 2B).

The membrane conductance evoked by uncaged GABA desensitized to a few (1–3) levels of GABA channel openings within 30–90 s after the laser pulse (Figure 4A). GABA channels were recorded at different *Vp*-values and the amplitudes of currents via single GABA channels were plotted against the pipette potential (Figure 4B). To ensure that the voltage imposed on the pipette did not affect the membrane potential (Alcami et al., 2012) we controlled the firing rate of the recorded cells and did not observe any dependence in the action potential on *Vp*-values. An exponential fit of the current-voltage relationships of the current amplitudes through single GABA channels revealed an elementary conductance of 11.3 ± 0.3 pS in the range of *Vp*-values from 0 to −50 mV, and a reversal potential of −15.0 ± 1.7 mV, corresponding to *DF*_GABA_ of +15 mV (*n* = 3) that is close to previously reported values obtained in this age group during cell-attached recordings of single GABA channels activated by GABA continuously present in the pipette (Tyzio et al., 2006, 2008).

## Discussion

In the present study, we describe a novel technique to assess the polarity of responses to GABA using non-invasive cell-attached recordings of responses evoked by laser uncaging of GABA from RuBi-GABA. We demonstrate that intrapipette GABA uncaging evokes integral GABA(A) receptor mediated currents which show depolarizing polarity in immature neurons.

This technique has several advantages over conventional cell-attached recordings of GABA channels activated by GABA continuously present in the pipette. First, it avoids a problem of contamination of the response by channels other than GABA-activated channels that is always a concern in cell-attached recordings. Second, simultaneous activation of many GABA channels by GABA transients generated by GABA uncaging significantly improves the signal-to-noise ratio of responses at the resting membrane potential, where the currents via single channels are hardly detectable from the baseline. This enables to conclude on the polarity of GABA responses directly from the direction of the initial response slope, while with single-channel recordings, the polarity of the GABA response often, particularly when the reversal potential of the currents via GABA channels is close to the resting membrane potential, can only be estimated from fits of the current-voltage relationships built from the currents recorded at potentials distant from the reversal potential.

Current-voltage relationships of the integral responses evoked by GABA uncaging also showed a depolarizing direction of GABA responses, as they reversed at potentials more positive than the resting membrane potential. Moreover, the reversal potential of the integral currents measured during ramp-protocols negatively shifted after the blockade of NKCC1 with bumetanide. This is in keeping with previous reports pointing out the pivotal role of NKCC1 in maintaining elevated intracellular chloride and depolarizing *DF*_GABA_ values in immature cortical neurons (Payne et al., 2003; Yamada et al., 2004; Dzhala et al., 2005; Tyzio et al., 2006; Brumback and Staley, 2008). Hysteresis of GABA responses (dependence of their reversal potential on the prehistory of membrane potential) has been seen during two-directional voltage ramp protocols in our cell-attached recordings of the integral responses evoked by uncaged GABA. This indicates that voltage-dependent changes in the intracellular chloride concentration are highly dynamic and may occur as a result of chloride flow via tens of GABA channels [comparable with ~thirty channels activated during unitary synaptic events (Edwards et al., 1990)] within hundreds of milliseconds. This corresponds to the hypothesis that intracellular chloride concentration is dynamically modified during physiological or paroxysmal activities so that chloride accumulates in synapses previously activated during strong cell depolarization (e.g., during spike trains or epileptic population spikes) (Kaila et al., 1997; Fujiwara-Tsukamoto et al., 2003, 2010; Ilie et al., 2012; for reviews, Isomura et al., 2008; Lamsa et al., 2010; Raimondo et al., 2012). An additional factor contributing to the hysteresis of GABA responses could be a change in the chloride concentration at the tip of the recording electrode. Indeed, the spatial angle from the patch into the cell is much larger than to the pipette, or in other words, diffusion within the pipette is nearly one dimensional, whereas in the cell it is three dimensional, and this geometry makes intrapipette chloride concentration prone to changes. Therefore, the overall effect could be that the chloride concentration changes on both sides of the patch, and hysteresis is caused by both intra- and extracellular ionic shifts. In addition to hysteresis, in a number of cells we also observed action potentials generated at *Vp* = 0 after the initial response (Figure 2B) or at positive pipette potentials during ramp commands (Figure 2A) suggesting an electrical access to the recorded cell via a large number of GABA channels activated by uncaged GABA. Electrical access to the cell would affect the membrane potential and would contaminate cell-attached currents with whole-cell currents, thus introducing an additional source of error in the measurements of *DF*_GABA_. Therefore, we conclude that the initial slope of the response evoked by uncaged GABA, which shows a depolarizing direction of GABA currents in immature cortical neurons that reverses to a hyperpolarizing direction after the blockade of NKCC1, and which shows a voltage-dependence comparable to that of the currents through single channels, is more reliable than voltage-ramp measurements to determine whether the recorded compartment of a cell is depolarized or hyperpolarized by GABA.

Thus, a novel method of cell-attached recordings of the responses evoked by uncaged GABA presented here can be reliably used to characterize the polarity of GABA responses in an unperturbed neuron. Present results were obtained using blind-patch recordings presumably (given the pipette resistance of 6–8 MOhm) from the soma of the immature neurons. With visual patch-clamp recordings, this technique can be particularly suited to study the actions of GABA in small cell compartments, e.g., in the initial axon segment or dendrites. It could be also used in a number of applications where the polarity of GABA responses is a subject of investigation, including development, activity-dependent alterations in the intracellular chloride, pathologies including epilepsy, trauma, and pain, as well as for a screening of molecules that modify GABA actions via alterations of intracellular chloride homeostasis.

## Author contributions

Rustem Khazipov and Marat Minlebaev conceived the project and designed experiments. Rustem Khazipov, Marat Minlebaev, Vadim Cheremizkin and Gaëlle Coustillier developed a system for the intrapipette laser uncaging. Rustem Khazipov, Marat Minlebaev, and Guzel Valeeva performed the experiments and analyzed the data. Rustem Khazipov wrote the paper.

### Conflict of interest statement

The authors declare that the research was conducted in the absence of any commercial or financial relationships that could be construed as a potential conflict of interest.

## References

[B1] Abe Y. Furukawa K. Itoyama Y. Akaike N. (1994). Glycine response in acutely dissociated ventromedial hypothalamic neuron of the rat: new approach with gramicidin perforated patch-clamp technique. J. Neurophysiol. 72, 1530–1537 7529820 10.1152/jn.1994.72.4.1530

[B2] Alcami P. Franconville R. Llano I. Marty A. (2012). Measuring the firing rate of high-resistance neurons with cell-attached recording. J. Neurosci. 32, 3118–3130 10.1523/JNEUROSCI.5371-11.2012 22378885 PMC6622012

[B3] Bazelot M. Dinocourt C. Cohen I. Miles R. (2010). Unitary inhibitory field potentials in the CA3 region of rat hippocampus. J. Physiol. 588, 2077–2090 10.1113/jphysiol.2009.185918 20403979 PMC2911213

[B4] Ben Ari Y. Gaiarsa J. L. Tyzio R. Khazipov R. (2007). GABA: a Pioneer transmitter that excites immature neurons and generates primitive oscillations. Physiol. Rev. 87, 1215–1284 10.1152/physrev.00017.2006 17928584

[B5] Blaesse P. Airaksinen M. S. Rivera C. Kaila K. (2009). Cation-chloride cotransporters and neuronal function. Neuron 61, 820–838 10.1016/j.neuron.2009.03.003 19323993

[B6] Boulenguez P. Liabeuf S. Bos R. Bras H. Jean-Xavier C. Brocard C. (2010). Down-regulation of the potassium-chloride cotransporter KCC2 contributes to spasticity after spinal cord injury. Nat. Med. 16, 302–307 10.1038/nm.2107 20190766

[B7] Brumback A. C. Staley K. J. (2008). Thermodynamic regulation of NKCC1-mediated Cl- cotransport underlies plasticity of GABA(A) signaling in neonatal neurons. J. Neurosci. 28, 1301–1312 10.1523/JNEUROSCI.3378-07.2008 18256250 PMC6671583

[B8] Chavas J. Marty A. (2003). Coexistence of excitatory and inhibitory GABA synapses in the cerebellar interneuron network. J. Neurosci. 23, 2019–2031 12657660 10.1523/JNEUROSCI.23-06-02019.2003 PMC6742031

[B9] Cherubini E. Gaïarsa J. -L. Ben-Ari Y. (1991). GABA: an excitatory transmitter in early postnatal life. Trends Neurosci. 14, 515–519 10.1016/0166-2236(91)90003-D 1726341

[B10] Cohen I. Navarro V. Clemenceau S. Baulac M. Miles R. (2002). On the origin of interictal activity in human temporal lobe epilepsy *in vitro*. Science 298, 1418–1421 10.1126/science.1076510 12434059

[B11] Connor J. A. Tseng H. Y. Hockberger P. E. (1987). Depolarization- and transmitter-induced changes in intracellular Ca2+ of rat cerebellar granular cells in explant cultures. J. Neurosci. 7, 1384–1400 2883269 10.1523/JNEUROSCI.07-05-01384.1987 PMC6568833

[B12] Curmi J. P. Premkumar L. S. Birnir B. Gage P. W. (1993). The influence of membrane potential on chloride channels activated by GABA in rat cultured hippocampal neurons. J. Membr. Biol. 136, 273–280 10.1007/BF00233666 8114077

[B13] De Koninck Y. (2007). Altered chloride homeostasis in neurological disorders: a new target. Curr. Opin. Pharmacol. 7, 93–99 10.1016/j.coph.2006.11.005 17182282

[B14] Dzhala V. Valeeva G. Glykys J. Khazipov R. Staley K. (2012). Traumatic alterations in GABA signaling disrupt hippocampal network activity in the developing brain. J. Neurosci. 32, 4017–4031 10.1523/JNEUROSCI.5139-11.2012 22442068 PMC3333790

[B15] Dzhala V. I. Kuchibhotla K. V. Glykys J. C. Kahle K. T. Swiercz W. B. Feng G. (2010). Progressive NKCC1-dependent neuronal chloride accumulation during neonatal seizures. J. Neurosci. 30, 11745–11761 10.1523/JNEUROSCI.1769-10.2010 20810895 PMC3070296

[B16] Dzhala V. I. Talos D. M. Sdrulla D. A. Brumback A. C. Mathews G. C. Benke T. A. (2005). NKCC1 transporter facilitates seizures in the developing brain. Nat. Med. 11, 1205–1213 10.1038/nm1301 16227993

[B17] Edwards F. A. Konnerth A. Sakmann B. (1990). Quantal analysis of inhibitory synaptic transmission in the dentate gyrus of rat hippocampal slices: a patch-clamp study. J. Physiol. (Lond.) 430, 213–249 1707966 10.1113/jphysiol.1990.sp018289 PMC1181735

[B18] Farrant M. Kaila K. (2007). The cellular, molecular and ionic basis of GABA(A) receptor signalling. Prog. Brain Res. 160, 59–87 10.1016/S0079-6123(06)60005-8 17499109

[B19] Freund T. Buzsaki G. (1996). Interneurons of the hippocampus. Hippocampus 6, 345–470 10.1002/(SICI)1098-1063(1996)6:4<347::AID-HIPO1>3.0.CO;2-I 8915675

[B20] Fujiwara-Tsukamoto Y. Isomura Y. Imanishi M. Ninomiya T. Tsukada M. Yanagawa Y. (2010). Prototypic seizure activity driven by mature hippocampal fast-spiking interneurons. J. Neurosci. 30, 13679–13689 10.1523/JNEUROSCI.1523-10.2010 20943908 PMC6633708

[B21] Fujiwara-Tsukamoto Y. Isomura Y. Nambu A. Takada M. (2003). Excitatory GABA input directly drives seizure-like rhythmic synchronization in mature hippocampal CA1 pyramidal cells. Neuroscience 119, 265–275 10.1016/S0306-4522(03)00102-7 12763087

[B22] Gaiarsa J. L. Tseeb V. Ben Ari Y. (1995). Postnatal development of pre- and postsynaptic GABAB-mediated inhibitions in the CA3 hippocampal region of the rat. J. Neurophysiol. 73, 246–255 7714569 10.1152/jn.1995.73.1.246

[B23] Glickfeld L. L. Roberts J. D. Somogyi P. Scanziani M. (2009). Interneurons hyperpolarize pyramidal cells along their entire somatodendritic axis. Nat. Neurosci. 12, 21–23 10.1038/nn.2230 19029887 PMC3505023

[B24] Glykys J. Dzhala V. I. Kuchibhotla K. V. Feng G. Kuner T. Augustine G. (2009). Differences in cortical versus subcortical GABAergic signaling: a candidate mechanism of electroclinical uncoupling of neonatal seizures. Neuron 63, 657–672 10.1016/j.neuron.2009.08.022 19755108 PMC2932871

[B25] Gulledge A. T. Stuart G. J. (2003). Excitatory actions of GABA in the cortex. Neuron 37, 299–309 10.1016/S0896-6273(02)01146-7 12546824

[B26] Huguenard J. R. Alger B. E. (1986). Whole-cell voltage-clamp study of the fading of GABA-Activated currents in acutely dissociated hippocampal neurons. J. Neurophysiol. 56, 1–18 3746390 10.1152/jn.1986.56.1.1

[B27] Ilie A. Raimondo J. V. Akerman C. J. (2012). Adenosine release during seizures attenuates GABAA receptor-mediated depolarization. J. Neurosci. 32, 5321–5332 10.1523/JNEUROSCI.5412-11.2012 22496577 PMC6622092

[B28] Isomura Y. Fujiwara-Tsukamoto Y. Takada M. (2008). A network mechanism underlying hippocampal seizure-like synchronous oscillations. Neurosci. Res. 61, 227–233 10.1016/j.neures.2008.04.002 18457889

[B29] Kaila K. Lamsa K. Smirnov S. Taira T. Voipio J. (1997). Long-lasting GABA-mediated depolarization evoked by high-frequency stimulation in pyramidal neurons of rat hippocampal slice is attributable to a network-driven, bicarbonate-dependent K+ transient. J. Neurosci. 17, 7662–7672 9315888 10.1523/JNEUROSCI.17-20-07662.1997 PMC6793904

[B30] Kakazu Y. Akaike N. Komiyama S. Nabekura J. (1999). Regulation of intracellular chloride by cotransporters in developing lateral superior olive neurons. J. Neurosci. 19, 2843–2851 10191302 10.1523/JNEUROSCI.19-08-02843.1999 PMC6782270

[B31] Khalilov I. Holmes G. L. Ben Ari Y. (2003). *In vitro* formation of a secondary epileptogenic mirror focus by interhippocampal propagation of seizures. Nat. Neurosci. 6, 1079–1085 10.1038/nn1125 14502289

[B32] Khazipov R. Leinekugel X. Khalilov I. Gaïarsa J. -L. Ben-Ari Y. (1997). Synchronization of GABAergic interneuronal network in CA3 subfield of neonatal rat hippocampal slices. J. Physiol. (Lond.) 498, 763–772 9051587 10.1113/jphysiol.1997.sp021900 PMC1159192

[B33] Khirug S. Yamada J. Afzalov R. Voipio J. Khiroug L. Kaila K. (2008). GABAergic depolarization of the axon initial segment in cortical principal neurons is caused by the Na-K-2Cl cotransporter NKCC1. J. Neurosci. 28, 4635–4639 10.1523/JNEUROSCI.0908-08.2008 18448640 PMC6670448

[B34] Kuner T. Augustine G. J. (2000). A genetically encoded ratiometric indicator for chloride: capturing chloride transients in cultured hippocampal neurons. Neuron 27, 447–459 10.1016/S0896-6273(00)00056-8 11055428

[B35] Lamsa K. P. Kullmann D. M. Woodin M. A. (2010). Spike-timing dependent plasticity in inhibitory circuits. Front. Synaptic Neurosci. 2:8 10.3389/fnsyn.2010.00008 21423494 PMC3059674

[B36] Leinekugel X. Medina I. Khalilov I. Ben-Ari Y. Khazipov R. (1997). Ca^2+^ oscillations mediated by the synergistic excitatory actions of GABA_A_ and NMDA receptors in the neonatal hippocampus. Neuron 18, 243–255 10.1016/S0896-6273(00)80265-2 9052795

[B37] Marandi N. Konnerth A. Garaschuk O. (2002). Two-photon chloride imaging in neurons of brain slices. Pflugers Arch. 445, 357–365 10.1007/s00424-002-0933-7 12466938

[B38] Martina M. Royer S. Pare D. (2001). Cell-type-specific GABA responses and chloride homeostasis in the cortex and amygdala. J. Neurophysiol. 86, 2887–2895 11731545 10.1152/jn.2001.86.6.2887

[B39] Marty A. Llano I. (2005). Excitatory effects of GABA in established brain networks. Trends Neurosci. 28, 284–289 10.1016/j.tins.2005.04.003 15927683

[B40] Mazzuca M. Minlebaev M. Shakirzyanova A. Tyzio R. Taccola G. Janackova S. (2011). Newborn Analgesia Mediated by Oxytocin during Delivery. Front. Cell. Neurosci. 5:3 10.3389/fncel.2011.00003 21519396 PMC3080614

[B41] Nabekura J. Ueno T. Okabe A. Furuta A. Iwaki T. Shimizu-Okabe C. (2002). Reduction of KCC2 expression and GABAA receptor-mediated excitation after *in vivo* axonal injury. J. Neurosci. 22, 4412–4417 12040048 10.1523/JNEUROSCI.22-11-04412.2002 PMC6758784

[B42] Owens D. F. Kriegstein A. R. (2002). Is there more to gaba than synaptic inhibition? Nat. Rev. Neurosci. 3, 715–727 10.1038/nrn919 12209120

[B43] Payne J. A. Rivera C. Voipio J. Kaila K. (2003). Cation-chloride co-transporters in neuronal communication, development and trauma. Trends Neurosci. 26, 199–206 10.1016/S0166-2236(03)00068-7 12689771

[B44] Perkins K. L. (2006). Cell-attached voltage-clamp and current-clamp recording and stimulation techniques in brain slices. J. Neurosci. Methods 154, 1–18 10.1016/j.jneumeth.2006.02.010 16554092 PMC2373773

[B45] Pond B. B. Berglund K. Kuner T. Feng G. Augustine G. J. Schwartz-Bloom R. D. (2006). The chloride transporter Na(+)-K(+)-Cl- cotransporter isoform-1 contributes to intracellular chloride increases after *in vitro* ischemia. J. Neurosci. 26, 1396–1406 10.1523/JNEUROSCI.1421-05.2006 16452663 PMC6675477

[B46] Price T. J. Cervero F. Gold M. S. Hammond D. L. Prescott S. A. (2009). Chloride regulation in the pain pathway. Brain Res. Rev. 60, 149–170 10.1016/j.brainresrev.2008.12.015 19167425 PMC2903433

[B47] Raimondo J. V. Markram H. Akerman C. J. (2012). Short-term ionic plasticity at GABAergic synapses. Front. Synaptic Neurosci. 4:5 10.3389/fnsyn.2012.00005 23087642 PMC3472547

[B48] Reichling D. B. Kyrozis A. Wang J. MacDermott A. B. (1994). Mechanisms of GABA and glycine depolarization-induced calcium transients in rat dorsal horn neurons. J. Physiol. (Lond.) 476, 411–421 8057250 10.1113/jphysiol.1994.sp020142 PMC1160455

[B49] Rial Verde E. M. Zayat L. Etchenique R. Yuste R. (2008). Photorelease of GABA with visible light using an inorganic caging group. Front. Neural Circuits 2:2 10.3389/neuro.04.002.2008 18946542 PMC2567106

[B50] Rivera C. Voipio J. Payne J. A. Ruusuvuori E. Lahtinen H. Lamsa K. (1999). The K+/Cl- co-transporter KCC2 renders GABA hyperpolarizing during neuronal maturation. Nature 397, 251–255 10.1038/16697 9930699

[B51] Serafini R. Valeyev A. Y. Barker J. L. Poulter M. O. (1995). Depolarizing GABA-activated Cl^−^ channels in embryonic rat spinal and olfactory bulb cells. J. Physiol. (Lond.) 488, 371–386 8568677 10.1113/jphysiol.1995.sp020973 PMC1156677

[B52] Shulga A. Thomas-Crusells J. Sigl T. Blaesse A. Mestres P. Meyer M. (2008). Posttraumatic GABA(A)-mediated [Ca2+]i increase is essential for the induction of brain-derived neurotrophic factor-dependent survival of mature central neurons. J. Neurosci. 28, 6996–7005 10.1523/JNEUROSCI.5268-07.2008 18596173 PMC6670975

[B53] Szabadics J. Varga C. Molnar G. Olah S. Barzo P. Tamas G. (2006). Excitatory effect of GABAergic axo-axonic cells in cortical microcircuits. Science 311, 233–235 10.1126/science.1121325 16410524

[B54] Thompson S. M. Gähwiler B. H. (1989). Activity-dependent disinhibition. I. Repetitive stimulation reduces IPSP driving force and conductance in the hippocampus *in vitro*. J. Neurophysiol. 61, 501–511 2709096 10.1152/jn.1989.61.3.501

[B55] Toyoda H. Ohno K. Yamada J. Ikeda M. Okabe A. Sato K. (2003). Induction of NMDA and GABAA receptor-mediated Ca2+ oscillations with KCC2 mRNA downregulation in injured facial motoneurons. J. Neurophysiol. 89, 1353–1362 10.1152/jn.00721.2002 12612004

[B56] Tyzio R. Cossart R. Khalilov I. Minlebaev M. Hubner C. A. Represa A. (2006). Maternal oxytocin triggers a transient inhibitory switch in GABA signaling in the fetal brain during delivery. Science 314, 1788–1792 10.1126/science.1133212 17170309

[B57] Tyzio R. Minlebaev M. Rheims S. Ivanov A. Jorquera I. Holmes G. L. (2008). Postnatal changes in somatic gamma-aminobutyric acid signalling in the rat hippocampus. Eur. J. Neurosci. 27, 2515–2528 10.1111/j.1460-9568.2008.06234.x 18547241

[B58] Valeeva G. Abdullin A. Tyzio R. Skorinkin A. Nikolski E. Ben-Ari Y. (2010). Temporal coding at the immature depolarizing GABAergic synapse. Front. Cell. Neurosci. 4 10.3389/fncel.2010.00017 20725525 PMC2914581

[B59] van den Pol A. N. Obrietan K. Chen G. (1996). Excitatory actions of GABA after neuronal trauma. J. Neurosci. 16, 4283–4292 8753889 10.1523/JNEUROSCI.16-13-04283.1996 PMC6578987

[B60] Verheugen J. A. Fricker D. Miles R. (1999). Noninvasive measurements of the membrane potential and GABAergic action in hippocampal interneurons. J. Neurosci. 19, 2546–2555 10087068 10.1523/JNEUROSCI.19-07-02546.1999 PMC6786065

[B61] Wei B. Kumada T. Furukawa T. Inoue K. Watanabe M. Sato K. (2013). Pre- and post-synaptic switches of GABA actions associated with Cl- homeostatic changes are induced in the spinal nucleus of the trigeminal nerve in a rat model of trigeminal neuropathic pain. Neuroscience 228, 334–348 10.1016/j.neuroscience.2012.10.043 23103796

[B62] Woodruff A. R. McGarry L. M. Vogels T. P. Inan M. Anderson S. A. Yuste R. (2011). State-dependent function of neocortical chandelier cells. J. Neurosci. 31, 17872–17886 10.1523/JNEUROSCI.3894-11.2011 22159102 PMC4071969

[B63] Yamada J. Okabe A. Toyoda H. Kilb W. Luhmann H. J. Fukuda A. (2004). Cl- uptake promoting depolarizing GABA actions in immature rat neocortical neurones is mediated by NKCC1. J. Physiol. (Lond.) 557, 829–841 10.1113/jphysiol.2004.062471 15090604 PMC1665166

[B64] Zhang S. J. Jackson M. B. (1995). GABAA receptor activation and the excitability of nerve terminals in the rat posterior pituitary. J. Physiol. (Lond.) 483, 583–595 7776245 10.1113/jphysiol.1995.sp020608 PMC1157804

